# Performance of swallowing function between older people with and without clinical complaints

**DOI:** 10.1590/2317-1782/e20240091en

**Published:** 2025-02-10

**Authors:** Ramon Cipriano Pacheco de Araújo, Cynthia Meira de Almeida Godoy, Lidiane Maria de Brito Macedo Ferreira, Juliana Fernandes Godoy, Hipólito Magalhães

**Affiliations:** 1 Programa Associado de Pós-graduação em Fonoaudiologia, Universidade Federal do Rio Grande do Norte – UFRN - Natal (RN), Brasil.; 2 Hospital Universitário Onofre Lopes – HUOL, Universidade Federal do Rio Grande do Norte – UFRN - Natal (RN), Brasil.; 3 Departamento de Fonoaudiologia, Universidade Federal do Rio Grande do Norte – UFRN - Natal (RN), Brasil.

**Keywords:** Aged, Deglutition Disorders, Deglutition, Pharynx, Elderly Nutrition

## Abstract

**Purpose:**

To compare the findings of speech-language-hearing evaluations, signs in fiberoptic endoscopic evaluation of swallowing, and nutritional risk between healthy older adults with and without self-reported swallowing difficulties and correlate the level of oral intake with the severity of pharyngeal residues and nutritional risk.

**Methods:**

This cross-sectional retrospective study included 71 older people and divided them into two groups based on the presence of swallowing complaints. Data were collected from speech-language-hearing evaluations, oral health status, and videoendoscopy signs with four food consistencies classified by the International Dysphagia Diet Standardisation Initiative (IDDSI) to compare the groups. Pharyngeal residues were analyzed and classified using the Yale Pharyngeal Residue Severity Rating Scale (YPRSRS), the level of oral intake was assessed using the Functional Oral Intake Scale (FOIS), and nutritional risk was evaluated using the Malnutrition Screening Tool (MST).

**Results:**

Differences were found in speech-language-hearing evaluations, as well as signs of posterior oral spillage and pharyngeal residues with levels 0, 2, and 4 consistencies and laryngeal penetration with level 0 consistency. The level of oral intake was moderately negatively correlated with the severity of pharyngeal residues and nutritional risk.

**Conclusion:**

The group of older adults with complaints had differences in speech-language-hearing evaluations, posterior oral spillage, and pharyngeal residues with levels 0, 2, and 4 consistencies, and laryngeal penetration with level 0 consistency. The correlation indicated that the lower the level of oral intake, the greater the severity of pharyngeal residues and nutritional risk in the sample.

## INTRODUCTION

Morphology gradually changes and physiological functions decline with aging across various body systems and regions^(1)^. The process of functional degradation is mediated by characteristic cellular and molecular functions associated with healthy aging^(1)^. This phenomenon can be divided into three categories: primary, antagonistic, and integrative characteristics^(2)^. Hence, when tissue-related homeostasis cannot compensate for the cumulative damage caused by primary and antagonistic characteristics, integrative characteristics prevail and ultimately lead to age-related functional decline^(2)^.

Cellular senescence occurs with advancing age through the accumulation of chondrocytes, which produce a factor that inhibits cartilage regeneration^(3)^. Aging cartilage weakens, has its shape changed, and loses functional elasticity^(4)^. Changes also take place in the musculoskeletal complex, with an annual 0.5% to 1.0% decrease in total muscle mass, escalating to a loss of up to 50% after the age of 80, along with a reduction in muscle contraction strength^(5)^. Consequently, healthy older adults are defined as those who experience age-related senescent changes and comorbidities but have no history of neurological or mechanical impairments^(2)^. Such deterioration is also one of the main contributors to the risk of oropharyngeal dysphagia in older adults.

Age-related changes in swallowing performance are influenced by several factors, such as tooth loss, reduced intraoral sensitivity, and significant musculoskeletal alterations^(4)^. However, their impact on the safety and efficiency of swallowing remains ambiguous. Some studies report an increase in episodes of laryngeal penetration and pharyngeal residue in supposedly healthy older adults^(1,6)^, while other ones report no differences in instrumental findings^(7,8)^. In addition to the conflicting results, there are also methodological differences, sample size limitations, and age range restrictions, which prevent the generalization of their findings^(4,9)^.

Moreover, recent studies have linked other factors, such as cognitive function, to dysphagia in aging, as evidence suggests that variations in neural activity and reorganization occur in older adults as a compensatory response to aging^(10)^. Although compensatory changes require greater effort during swallowing, these findings may impair the ability to adequately protect the lower airways and negatively impact the quality of life^(11)^. Therefore, these age-related changes can lead to various clinical practices to distinguish typical from atypical impairments, as well as individual clinical characteristics, which may be mistakenly classified as presbyphagia.

To address this issue, we hypothesized that older adults with clinical complaints of dysphagia, who appear otherwise healthy, may have different subjective and objective swallowing findings from those without clinical complaints but whose evaluation might still indicate changes. Thus, this study aimed to compare speech-language-hearing (SLH) assessment findings, signs in fiberoptic endoscopic evaluations of swallowing (FEES), and nutritional risk between healthy older adults with and without clinical complaints of swallowing difficulty. It also aimed to correlate the level of oral intake with the severity of pharyngeal residue and nutritional risk.

## METHODS

This cross-sectional retrospective study was based on data collected from medical records. The research was conducted at the otorhinolaryngology outpatient clinic of Onofre Lopes University Hospital (HUOL), collecting FEES data performed on patients between 2015 and 2023. All participants or their legal guardians signed an informed consent form previously provided by the service before the examination procedures. The study was approved by the HUOL Research Ethics Committee, under evaluation report number 6.169.294. The collected data included medical history, previous SLH assessments, FEES findings, and post-examination nutritional screening.

### Sample

The sample consisted of 71 older adults selected by convenience from individuals seeking care at the specified location. They were divided into two groups based on the presence of swallowing difficulty complaints. None of the participants had undergone SLH before the study. The first group comprised 44 individuals aged 60 to 94 years, predominantly females (63.6%), who reported idiopathic swallowing difficulties. These complaints were identified by other healthcare professionals at the facility and/or through referrals from other hospital departments where they also had consultations for instrumental examination, without using a specific dysphagia screening protocol. Exclusion criteria for this group were neurological diagnoses, inability to follow commands, history of cancer treatment, tracheostomy, alternative feeding routes, and orotracheal intubation within 12 months of the examination.

The second group had 27 individuals aged 60 to 79 years, with a predominance of females (59.2%), who did not report complaints of swallowing difficulties. Volunteers were recruited for the study in the waiting room of the service's consultation area with a simple self-perception question about swallowing difficulty. Exclusion criteria for this group were neurological diagnoses, history of cancer treatment, tracheostomy, alternative feeding routes, atypical psychiatric disorders, inability to follow commands, and history of orotracheal intubation within 12 months of the examination.

### Procedures

The clinical SLH evaluation was conducted before the instrumental examination when the patient was admitted to the outpatient clinic. An SLH pathologist from the service, with expertise in oropharyngeal dysphagia, performed the procedures. This evaluation used a protocol specific to the service, which analyzed orofacial myofunctional aspects, oral condition, phonation function, and cough efficiency.

The oral status was assessed by evaluating oral rehabilitation, salivary stasis, and the distribution of occlusal molar support, classified according to the Eichner Index (EI)^(12)^. This tooth loss classification method is based on occlusal contact between existing teeth in the premolar and molar regions. The EI was determined by the vertical contact components between the bilateral molars and categorized into three types: Class A, contact between four occlusal support zones; Class B, contact between one to three occlusal support zones; and Class C, no occlusal contact. Besides describing denture use, the EI was assessed using the habitual chewing occlusal support (i.e., using the current prosthetic rehabilitation).

The SLH pathologist subjectively assessed tongue mobility and strength, asking the patient to perform desired movements of tongue protrusion, lateralization, and protrusion against the resistance of a gloved finger. Normality criteria were defined as the ability to execute the desired commands correctly and maintain isometric strength against the finger's resistance. Tongue weakness was noted when the evaluator asked the patient to use maximum voluntary tongue force against the gloved finger's resistance. Although these are qualitative measures, depending on the evaluator's prior experience in comparing normality, weakness leads to brief muscle contraction and a rapid decrease in isometric movement^(13)^.

Regarding phonation function, the evaluator asked the patient to produce the vowel sound “a” for as long as possible, following a model. Normality criteria were defined as a maximum phonation time (MPT) of 14 seconds for women and 20 seconds for men^(14)^. An auditory-perceptual evaluation of voice was performed at the same time, noting the presence or absence of roughness during vowel emission. The patient was also asked to produce a strong spontaneous cough to assess the subjective efficiency of cough production on command (efficient/weak) for potential pharyngeal clearance. All changes were described and recorded to proceed with the instrumental swallowing evaluation.

A medical resident performed the FEES supervised by an otorhinolaryngologist and accompanied by an SLH pathologist with experience in oropharyngeal dysphagia, in accordance with the institution's protocol. They used a flexible nasopharyngoscope, Olympus^®^ brand, 3.2 mm in diameter, model LF-P with an attached micro camera and light source. The patient was instructed to remain seated throughout the examination, using no topical anesthetic to insert the instrument into the nasal cavity up to the hypopharynx. Pharyngeal sensitivity was tested by touching the nasopharyngoscope to the epiglottic region to verify pharyngeal constriction responses. After the physician analyzed the structures, the SLH pathologist offered foods artificially flavored with powdered diet juice, artificially colored with blue dye, and thickened with an instant cornstarch product. At the end, an 8-g portion of salted crackers was offered at will.

The food consistencies were evaluated according to the International Dysphagia Diet Standardisation Initiative (IDDSI) classification^(15)^, in this order: level 2 (mildly thick liquid), level 4 (extremely thick liquid), and level 0 (thin liquid) in three 5 mL servings on a metal spoon, while level 7 (regular solid) was offered as a single portion.

These three professionals, with experience in conducting the examination, also interpreted and assessed simultaneously and by consensus the presence of multiple swallows, posterior oral spillage, pharyngeal residue in the vallecula and/or piriform sinuses – according to the Yale Pharyngeal Residue Severity Rating Scale (YPRSRS)^(16)^ (1 - None, 2 - Trace, 3 - Mild residue, 4 - Moderate residue, 5 - Severe residue) –, and laryngeal penetration and aspiration. They used the following parameters for analysis, starting with the first offer: multiple swallows, defined as more than two attempts to swallow the same offer^(17,18)^; posterior oral spillage, identified by the premature escape of food into the vallecular region before triggering the swallow reflex^(17,18)^; pharyngeal residue, identified by the presence of residual colored food in the vallecula and/or piriform sinuses after swallowing the first offer^(17,18)^; laryngeal penetration, observed by the presence of residual colored food in the vocal fold^(17,18)^; and laryngeal aspiration, presence of residual colored food below the vocal folds^(17,18)^. All analyses were conducted in real-time, and the images were stored on a computer at the outpatient clinic, allowing for review as many times as the professionals deemed necessary after the examination.

The professionals assessed the level of oral intake after the examination, using the Functional Oral Intake Scale (FOIS)^(19)^ based on the examination analysis and the existence and need for liquid thickening. FOIS scores range from 1 (nothing by mouth) to 7 (full oral intake without restrictions). A nutritionist from the service assessed nutritional risk by applying the Malnutrition Screening Tool (MST)^(20)^, translated and adapted into Portuguese, which consists of three questions on the self-perception of weight loss and loss of appetite in the previous month. The MST is an accessible and quick tool to apply to adults upon hospital admission, whose scores of two or higher indicate nutritional risk and the need for a detailed nutritional assessment.

### Data analysis

Descriptive statistics were used for data analysis, including measures of central tendency and dispersion, and absolute and relative frequencies. The Shapiro-Wilk test checked the normality of the distribution of the dependent quantitative variables. The Mann-Whitney test compared the protocols for inferential analysis, while Pearson's chi-square or Fisher's exact test analyzed categorical variables, such as “SLH evaluation” and “FEES pharyngeal signs,” depending on whether the expected frequency for each cell was greater than or equal to 5. Additionally, Spearman's bivariate correlation with simple linear regression was used to obtain the coefficient of determination between the quantitative variables in the sample. A 5% significance level was considered for all analyses.

## RESULTS

The sample was divided into two groups based on self-perception of swallowing complaints: the first group included 44 older individuals with complaints of swallowing difficulty, with a mean age of 71.5 (±8.7) years, and the second group included 27 older individuals without complaints of swallowing difficulty, with a mean age of 67.9 (±5.3) years. Table 1 describes the characteristics and comparisons of the groups, such as sex, age, systemic arterial hypertension, diabetes mellitus, and polypharmacy. There was no difference in diagnoses or polypharmacy between the groups.

**Table 1:** Comparison between sex, age, systemic arterial hypertension, diabetes mellitus, and polypharmacy between groups

Variables	Groups		p-value
	Older people with complaints	Older people without complaints	
	n = 44 (%)	n = 27 (%)	
Sex			
Females	28 (63.6)	16 (59.3)	0.712
Males	16 (36.4)	11 (40.7)	
Age (years)	71.5 (±8.71)	67.9 (±5.39)	-
Systemic arterial hypertension			
Yes	14 (31.8)	8 (29.6)	0.846
No	30 (68.2)	19 (70.4)	
Diabetes Mellitus			
Yes	5 (11.4)	3 (11.1)	0.944
No	39 (88.6)	24 (88.9)	
Polypharmacy			
Yes	8 (18.2)	3 (11.1)	0.424
No	36 (81.8)	24 (88.9)	

All data are expressed as numbers (%) or means (standard deviations)

Table 2 presents the SLH evaluation findings between the groups. Older individuals with complaints had differences, such as reduced tongue strength, reduced MPT, and hoarseness during vowel emission. Regarding oral status, the group of older individuals with complaints had differences in denture use and habitual occlusion classified as EI class B or C, compared to the group without complaints.

**Table 2:** Comparison between speech-language-hearing findings and oral status between groups

Speech-language-hearing findings	Groups		p-value
	Older people with complaints	Older people without complaints	
	n= 44 (%)	n= 27 (%)	
Tongue mobility			
Preserved	35 (79.5)	25 (92.6)	0.187
Reduced	9 (20.5)	2 (7.4)	
Tongue strength			
Preserved	30 (68.2)	25 (92.6)	**0.020** **
Reduced	14 (31.8)	2 (7.4)	
Oral transit time			
Adequate	41 (93.2)	26 (96.3)	0.581
Increased	3 (6.8)	1 (3.7)	
Maximum phonation time			
Adequate	32 (72.7)	25 (11.1)	0.050**
Reduced	12 (27.3)	2 (88.9)	
Roughness			
Absent	20 (45.5)	20 (74.1)	**0.018** *
Present	24 (54.5)	7 (25.9)	
Spontaneous cough			
Efficient	37 (84.1)	26 (96.3)	0.114
Weak	7 (15.9)	1 (3.7)	
**Oral status**			
Denture use Yes No	8 (18.2) 36 (81.8)	12 (44.4) 15 (55.6)	0.017*
Eichner Index Class A Class B or C	23 (52.3) 21 (47.7)	21 (77.8) 6 (22.2)	0.032*
Salivary stasis Absent Present	42 (95.5) 2 (4.5)	27 (100) 0 (0.0)	**0.522**

* Pearson’s chi-square;

** Fisher’s exact

All data are expressed as numbers (%)

The relationship of FEES pharyngeal signs, presented in Table 3, shows differences in the occurrences of posterior oral spillage and pharyngeal residue with levels 0, 2, and 4 consistencies. There was also a difference in the occurrences of laryngeal penetration with level 0 consistency. The group of older adults with complaints was the only one with signs of laryngeal penetration and aspiration with thick liquids (levels 2 and 4). No pharyngeal signs were found with the regular solid consistency (level 7) in the group of older individuals without complaints.

**Table 3:** Relationship between pharyngeal signs of videoendoscopy of swallowing by food consistency between groups

Pharyngeal signs per food consistency level (IDDSI)	Groups		p-value
	Older people with complaints	Older people without complaints	
	n= 44 (%)	n= 27 (%)	
Pharyngeal sensitivity			
Preserved	36 (81.8)	24 (88.9)	0.515
Reduced	8 (18.2)	3 (11.1)	
Glottal closure			
Complete	40 (90.9)	27 (100)	0.290
Incomplete	4 (9.1)	0 (0.0)	
Thin liquid (level 0)			
Multiple swallows			
Absent	41 (93.2)	26 (96.3)	0.581
Present	3 (6.8)	1 (3.7)	
Posterior oral spillage			
Absent	21 (47.7)	20 (74.1)	0.029*
Present	23 (52.3)	7 (25.9)	
Pharyngeal residues			
Absent	21 (47.7)	20 (74.1)	**0.029** *
Present	23 (52.3)	7 (25.9)	
Laryngeal penetration			
Absent	37 (84.1)	27 (100)	**0.039** **
Present	7 (15.9)	0 (0.0)	
Laryngeal aspiration			
Absent	42 (95.5)	27 (100)	0.522
Present	2 (4.5)	0 (0.0)	
Mildly thick liquid (level 2)			
Multiple swallows			
Absent	41 (93.2)	27 (100)	0.283
Present	3 (6.8)	0 (0.0)	
Posterior oral spillage			
Absent	22 (50.0)	21 (77.8)	0.020*
Present	22 (50.0)	6 (22.2)	
Pharyngeal residues			
Absent	24 (54.5)	22 (81.5)	0.021*
Present	20 (45.5)	5 (18.5)	
Laryngeal penetration			
Absent	41 (93.2)	27 (100)	0.283
Present	3 (6.8)	0 (0.0)	
Laryngeal aspiration			
Absent	43 (97.7)	27 (100)	0.430
Present	1 (2.3)	0 (0.0)	
Extremely thick liquid (level 4)			
Multiple swallows			
Absent	41 (93.2)	27 (100)	0.283
Present	3 (6.8)	0 (0.0)	
Posterior oral spillage			
Absent	23 (52.3)	23 (85.2)	0.005**
Present	21 (47.7)	4 (14.8)	
Pharyngeal residues			
Absent	22 (50.0)	24 (88.9)	<0.001**
Present	22 (50.0)	3 (11.1)	
Laryngeal penetration			
Absent	43 (97.7)	27 (100)	0.430
Present	1 (2.3)	0 (0.0)	
Laryngeal aspiration			
Absent	44 (100)	27 (100)	-
Present	0 (0.0)	0 (0.0)	
Regular solid (level 7)			
Multiple swallows			
Absent	44 (100)	27 (100)	-
Present	0 (0.0)	0 (0.0)	
Posterior oral spillage			
Absent	39 (88.6)	27 (100)	0.149
Present	5 (11.4)	0 (0.0)	
Pharyngeal residues			
Absent	38 (86.4)	27 (100)	0.076
Present	6 (13.6)	0 (0.0)	
Laryngeal penetration			
Absent	44 (100)	27 (100)	-
Present	0 (0.0)	0 (0.0)	
Laryngeal aspiration			
Absent	44 (100)	27 (100)	-
Present	0 (0.0)	0 (0.0)	

* Pearson’s chi-square;

** Fisher’s exact

Caption: IDDSI = International Dysphagia Diet Standardisation Initiative

All data are expressed as numbers (%)

When comparing the equality of the medians of the pharyngeal residue classification protocol (YPRSRS), oral intake level (FOIS), and nutritional risk (MST), the group of older individuals with complaints differed significantly from the group without complaints (Table 4). The group of older adults with complaints primarily had residues classified as trace level (YPRSRS 2), whereas the group without complaints had no residue (YPRSRS 1) in the vallecula and piriform sinuses.

**Table 4:** Comparison between the severity of pharyngeal residues, nutritional risk, and the level of oral intake between the groups

	Groups		p-value
	Older people with complaints	Older people without complaints	
YPRSRS	2 (1-3)	1 (1-1)	0.011*
MST	0 (0-1.2)	0 (0-0)	**0.009** *
FOIS	5.5 (5-6.2)	7 (7-7)	<0.001*

* Mann-Whitney U test

Caption: YPRSRS = Yale Pharyngeal Residue Severity Rating Scale; MST = Malnutrition Screening Tool; FOIS = Functional Oral Intake Scale

All data are expressed as medians (Q_1_-Q_3_ interquartile range)

Table 5 presents the results of the Spearman correlation analysis between the FOIS and the YPRSRS and MST in the sample – it moderately negatively correlated with both the YPRSRS and MST. The coefficient of determination indicated that the severity of pharyngeal residue and nutritional risk were responsible for 30% and 38%, respectively, of the influence on the oral intake level in the individuals of the sample. The formula for predicting the oral intake level can be represented by 7.15 * (-0.60) * (YPRSRS value); 6.39 * (-0.73) * (MST value).

**Table 5:** Correlation and linear regression between the level of oral intake and the severity of pharyngeal residues and nutritional risk in the sample

Severity of pharyngeal residues (YPRSRS) and nutritional risk (MST)	FOIS		
	⍴	p-value	R^2^
YPRSRS	-0.526	<0.001	0.305
MST	-0.622	<0.001	0.387

Caption: YPRSRS = Yale Pharyngeal Residue Severity Rating Scale; MST = Malnutrition Screening Tool; FOIS = Functional Oral Intake Scale; R^2^ = Coefficient of determination; Spearman correlation and simple linear regression

## DISCUSSION

The study results suggest that the group of older adults with complaints had differences, including reduced tongue strength, decreased MPT, and roughness in vocal emission. There were also occurrences of posterior oral spillage and pharyngeal residues with level 0, 2, and 4 consistencies and laryngeal penetration with level 0 consistency. The oral intake level was moderately negatively correlated with the severity of pharyngeal residues and nutritional risk in the sample.

Age-related functional changes in the oral phase have been previously investigated, with a focus on studies examining tongue mobility and strength, as the tongue plays a crucial role in capturing, applying pressure, transporting, and propelling the bolus from the oral cavity to the pharyngeal phase^(21)^. The study results suggest that tongue strength may be reduced for its swallowing tasks, which could, in turn, affect its efficiency, despite being measured before the activity. Therefore, this finding supports other studies that have identified age-related changes in tongue muscles, with a significant subsequent reduction in intraoral pressure^(21,22)^, although there is no data on the reduction of tongue activity during the task.

Phonation parameters were investigated to provide relevant information about the vocal fold closure and potential organic changes in the larynx that could reduce the ability to protect the lower airways during swallowing. The reduced MPT in the group of older adults with complaints may be related to a decreased ability to protect the airways and the presence of food penetration into the vocal folds, as evidenced with the thin liquid consistency (level 0). There is evidence that fibroblast activity and hyaluronic acid production decrease in older people^(9)^. As a result, vocal ligament stiffness increases, and the higher concentration of collagen reduces the viscoelastic properties of the mucosa, which may lead to roughness in the auditory-perceptual evaluation of voice^(9)^.

Concerning oral status, the group of older individuals with complaints had differences in denture use and occlusal contact between molar zones. The study group had less dentures use, corresponding to a higher presence of EI Class B or C. This means significant tooth losses in the occlusal contact zones between molars during habitual chewing, without the possibility of oral rehabilitation. The consequences of tooth loss in older adults have been widely researched and suggest that the number of missing teeth is related to reduced chewing efficiency, longer food processing time, and the selection of more chewable foods^(23)^. Moreover, the prevalence of root caries in individuals over 60 years old is twice as high as in younger people, which can make the pulp's neuromuscular structure sensitive, painful, and even prone to infections^(24)^. This can reduce the chewing and intake of vegetables, fruits, and fiber, consequently increasing the risk of significant weight loss and nutritional risk^(25)^.

Pharyngeal signs were analyzed through FEES with four different food consistencies. The group of older adults with complaints had differences in signs of posterior oral spillage and pharyngeal residue with liquids (levels 0, 2, and 4), while laryngeal penetration occurred with thin liquid (level 0). Since no differences were found in dysphagia signs with solid food, the more fluid-like consistencies (particularly non-thickened liquids) were the most unsafe for the study group. In another study, about 55% of older adults had signs of penetration with liquids during swallowing, in the same proportion of the reduction in the cough reflex^(26)^. Despite signs of impaired swallowing efficiency, the results suggest that the more severe signs of dysphagia were predominant in the group with complaints, as the recurrent pharyngeal residues and the possibility of eventual penetration of this material after swallowing may be predictors for clinical complaints^(27)^. If not effectively eliminated from the pharynx through protective responses like coughing or throat clearing, liquids can become a challenge in the person’s daily life.

The comparison of protocol medians showed differences in oral intake levels, pharyngeal residue grades, and nutritional risk between the groups. The severity of residue was significant for the group with complaints, who also had reduced tongue strength and laryngeal penetration in the same proportion. This demonstrates a chain of impairments in the progression of the swallowing phases, compared to the group without complaints. The presence of residue after swallowing and the severity of accumulation in the pharyngeal recesses are related to reduced tongue muscle activity and decreased hyolaryngeal movement, as observed in a previous study with dysphagic adults^(28)^. Thus, although most individuals in the group with complaints had no nutritional risk, the interquartile range was larger, indicating that some individuals were at nutritional risk due to swallowing impairments. Since the sample comprised community-dwelling older people who sought care, nutritional risk would be mitigated by dietary modifications and reduced intake – whereas in hospitalized older adults, nutritional risk represents 51%^(25,27)^.

The sample results indicated that the severity of residue and nutritional risk are correlated with and inversely proportional to oral intake. The lower the possibility of oral intake, the higher the degree of pharyngeal residue and nutritional risk. There is strong evidence that pharyngeal residue is a predictor for penetration and aspiration events in dysphagic patients^(28)^. However, thin and slightly thick liquids have higher frequencies of laryngeal penetration than other consistencies^(29)^. Although it may seem tempting to think that thick liquids are safer, it should be noted that the volume offered in the spoon (5 mL) could be a confounding factor between the assessment and the individual’s daily consumption.

Another factor to consider is the increase in nutritional risk as the level of oral intake decreases. By confirming that nutritional risk is related to swallowing disorders, we can hypothesize that the older people in the sample may restrict the consumption of certain foods due to difficulty in swallowing and experience eventual loss of appetite identified in nutritional screening. It is known that the processing and liquefaction of food without proper nutritional guidance can decrease caloric intake, nutritional status, and appetite^(30)^. Both malnutrition and dehydration can be caused by dysphagia, but they also worsen swallowing disorders^(30,31)^. In this cyclical effect, it is important to highlight the significance of monitoring and optimizing nutritional status for the proper management of dysphagia in older adults.

The limitations of the study include the absence of a validated dysphagia screening instrument for the group with complaints. However, given that the study participants were referred with symptoms to undergo objective swallowing investigation, this may negate the need for a screening tool. It is also important to mention the lack of instrumental tongue strength assessment, the unequal number of participants in each group, the absence of information regarding occupational history and exposure to chemical agents, and the lack of complementary data on nutritional status, such as food recall. The study's strengths are the careful sample selection, the subjective and instrumental analysis of swallowing, and the evaluation of oral intake levels and nutritional risk between the groups. These factors helped develop new research hypotheses regarding when swallowing difficulties become a complaint and provided new perspectives on dysphagia in seemingly healthy older individuals.

## CONCLUSION

The group of older adults with complaints had differences, including reduced tongue strength, shorter MPT, and roughness in voice emission. There were also occurrences of posterior oral spillage and pharyngeal residues with levels 0, 2, and 4 consistencies and laryngeal penetration with level 0 consistency. The correlation indicated that the lower the level of oral intake, the greater the severity of pharyngeal residues and the nutritional risk in the sample.
