# Supplementary material for: Performance of swallowing function between older people with and without clinical complaints
Source: Codas. 2025 Feb 10;37(2):e20240091. doi: 10.1590/2317-1782/e20240091en (PMC11813179; doi:10.1590/2317-1782/e20240091en)
Supplement: Translation

# Translation: Desempenho da função de deglutição entre idosos com e sem queixas clínicas

**DOI:** 10.1590/2317-1782/e20240091pt

**Authors:** Ramon Cipriano Pacheco de Araújo, Cynthia Meira de Almeida Godoy, Lidiane Maria de Brito Macedo Ferreira, Juliana Fernandes Godoy, Hipólito Magalhães

**Affiliations:** 1 Programa Associado de Pós-graduação em Fonoaudiologia, Universidade Federal do Rio Grande do Norte – UFRN - Natal (RN), Brasil.; 2 Hospital Universitário Onofre Lopes – HUOL, Universidade Federal do Rio Grande do Norte – UFRN - Natal (RN), Brasil.; 3 Departamento de Fonoaudiologia, Universidade Federal do Rio Grande do Norte – UFRN - Natal (RN), Brasil.

**Keywords:** Idoso, Transtornos de Deglutição, Deglutição, Faringe, Nutrição do Idoso

## Abstract

**Objetivo:**

Comparar os achados da avaliação fonoaudiológica, sinais da videoendoscopia da deglutição e o risco nutricional entre idosos saudáveis com e sem dificuldades autorreferidas em deglutir, além de correlacionar o nível de ingestão oral com a gravidade dos resíduos faríngeos e o risco nutricional.

**Método:**

Trata-se de um estudo transversal e retrospectivo. Foram incluídos 71 idosos divididos em dois grupos de acordo com a presença de queixas de deglutição. Foram coletados dados da avaliação fonoaudiológica e estado oral, além dos sinais videoendoscópios em quatro consistências alimentares classificados pelo International Dysphagia Diet Standartisation Initiative (IDDSI) para comparação entre os grupos. Os resíduos faríngeos foram analisados e classificados pelo Yale Pharyngeal Residue Severity Rating Scale (YPRSRS), o nível de ingestão oral pelo Functional Oral Intake Scale (FOIS) e o risco nutricional foi avaliado utilizando-se o Malnutrition Screening Tool (MST).

**Resultados:**

Houve diferença na avaliação fonoaudiológica, além de sinais de escape oral posterior e resíduos faríngeos nas consistências alimentares de nível 0, 2 e 4, e penetração laríngea na consistência de nível 0. Houve correlação negativa moderada entre o nível de ingestão oral, gravidade dos resíduos faríngeos e o risco nutricional.

**Conclusão:**

O grupo de idosos com queixas apresentou diferenças na avaliação fonoaudiológica, além de escape oral posterior e resíduos faríngeos nas consistências alimentares de nível 0, 2 e 4, e penetração laríngea na consistência de nível 0. A correlação indicou que, quanto menor o nível de ingestão oral, maior a gravidade dos resíduos faríngeos e o risco nutricional na amostra.

## INTRODUÇÃO

Durante o envelhecimento há uma modificação gradual na morfologia e declínio das funções fisiológicas em diversos sistemas e regiões corporais^(1)^. O processo de degradação funcional é mediado por funções celulares e moleculares característicos associados ao envelhecimento saudável^(1)^. Esse fenômeno pode ser dividido em três categorias, sendo características primárias, antagônicas e integrativas^(2)^. Nessa perspectiva, quando a homeostase relacionada ao tecido não pode compensar os danos cumulativos causados por características primárias e antagônicas, as características integrativas sobressaem e, por fim, levam ao declínio funcional relacionado à idade^(2)^.

A senescência celular pode ser observada no avançar da idade com acúmulos de condrócitos, que produzem um fator que inibe a regeneração das cartilagens^(3)^. A cartilagem envelhecida passa por mudanças na forma, enfraquecimento e perda de elasticidade durante sua funcionalidade^(4)^. Além disso, ocorrem mudanças no complexo musculoesquelético, com uma diminuição anual da massa muscular total de 0,5% a 1,0% e um aumento dessa perda de até 50% após os 80 anos de idade, assim como uma redução na força de contração muscular^(5)^. Em vista disso, entende-se que idosos saudáveis são aqueles que experimentam mudanças senescentes e comorbidades relacionadas à idade, mas sem histórico de comprometimentos neurológicos ou mecânicos^(2)^. Esse processo de deterioração também é um dos principais contribuintes para o risco de disfagia orofaríngea na população idosa.

As alterações no desempenho da deglutição com o envelhecimento são pressupostas por vários fatores, como ausências dentárias, redução na sensibilidade intraoral e alterações musculoesqueléticas relevantes^(4)^. No entanto, o impacto dessas mudanças na segurança e eficiência da deglutição são ambíguos. Alguns estudos relatam aumento de episódios de penetração laríngea e resíduos faríngeos em idosos supostamente saudáveis^(1,6)^, enquanto outros estudos relatam que não houve diferença nos achados instrumentais^(7,8)^. Nesse sentido, além de resultados divergentes, também há diferenças metodológicas, no tamanho amostral e limitação na faixa etária, que não permitem generalizações de seus resultados^(4,9)^.

Ademais, recentemente, outros fatores estão sendo relacionados à disfagia no envelhecimento como a função cognitiva, visto que as evidências sugerem que variações na atividade neural e reorganizações ocorrem em idosos como resposta compensatória ao envelhecimento^(10)^. Apesar das mudanças compensatórias exigirem um desempenho maior na deglutição, esses achados podem afetar a capacidade de proteger adequadamente as vias aéreas inferiores e repercutir de forma negativa na qualidade de vida^(11)^. Por isso, essas mudanças relacionadas à idade podem resultar em variações na prática clínica ao delinear modificações típicas ou atípicas do comprometimento, assim como particularidades clínicas do indivíduo que podem ser erroneamente classificados como presbifagia.

Para isso, levantamos uma hipótese que idosos com queixas clínicas de disfagia, aparentemente saudáveis, podem apresentar diferenças nos achados subjetivos e objetivos da deglutição, em comparação àqueles sem queixas clínicas, mas que podem estar suscetíveis a apresentar alterações na avaliação. Assim, o objetivo deste estudo foi comparar os achados da avaliação fonoaudiológica, sinais da videoendoscopia da deglutição e o risco nutricional entre idosos saudáveis com e sem queixas clínicas de dificuldade em deglutir, além de correlacionar o nível de ingestão oral com a gravidade dos resíduos faríngeos e o risco nutricional.

## MÉTODO

Trata-se de um estudo transversal e retrospectivo com base na coleta de dados dos prontuários. A pesquisa foi conduzida no ambulatório de otorrinolaringologia do Hospital Universitário Onofre Lopes (HUOL), em que foram coletados dados referentes ao exame da videoendoscopia da deglutição (VED) dos pacientes atendidos entre 2015 a 2023. Todos os participantes, ou responsáveis legais, assinaram o Termo de Consentimento Livre e Esclarecido (TCLE) disponibilizado previamente pelo serviço antes da realização dos procedimentos do exame. O estudo foi aprovado pelo Comitê de Ética em Pesquisa (CEP) do Hospital Universitário Onofre Lopes – CEP HUOL, sob parecer n° 6.169.294. Os dados coletados foram referentes ao histórico pregresso, avaliação fonoaudiológica prévia, achados da VED e a triagem nutricional pós exame.

### Casuística

A amostra foi constituída por 71 idosos escolhidos por conveniência entre os indivíduos que buscaram atendimento no referido local, em que foram divididos em dois grupos de acordo com a presença de queixas de dificuldade em deglutir. Nenhum participante da amostra realizou terapia fonoaudiológica previamente à pesquisa. O primeiro grupo foi composto por 44 indivíduos com idade entre 60 e 94 anos e com predomínio do gênero feminino (63,6%), que apresentavam queixas de dificuldade em deglutir por causa idiopática. As queixas foram identificadas por outros profissionais de saúde do serviço e/ou encaminhados por outros setores do próprio hospital em interconsultas, sem um protocolo específico de rastreamento de disfagia, para realizar o exame instrumental. Os critérios de exclusão nesse grupo foram diagnósticos neurológicos, incapazes de seguir comandos, histórico de tratamento oncológico, usuários de traqueostomia, via alternativa de alimentação e intubação orotraqueal nos últimos 12 meses prévios ao exame.

O segundo grupo foi composto por 27 indivíduos com idade entre 60 e 79 anos, com predomínio do gênero feminino (59,2%) e que não apresentavam queixas de dificuldade em deglutir. Dessa forma, a captação dos voluntários que aceitaram participar da pesquisa foi feita a partir de uma pergunta simples de autopercepção de dificuldade em deglutir, realizada na sala de espera de consultas do próprio serviço. Os critérios de exclusão aplicados nesse grupo foram diagnósticos neurológicos, histórico de tratamento oncológico, usuários de traqueostomia, via alternativa de alimentação, transtornos psiquiátricos atípicos, incapazes de seguir comandos e histórico de intubação orotraqueal nos últimos 12 meses prévios ao exame.

### Procedimentos

A avaliação clínica fonoaudiológica foi realizada previamente ao exame instrumental, no momento em que o paciente foi admitido no ambulatório. Um fonoaudiólogo do serviço com experiência em disfagia orofaríngea foi responsável por realizar os procedimentos. Nessa avaliação foi utilizado um protocolo próprio do serviço, em que foram analisados aspectos miofuncionais orofaciais, estado oral, função de fonação e eficiência na tosse.

O estado oral foi avaliado pelo uso de reabilitação oral, presença de estase salivar e a distribuição do suporte oclusal na região de molares, classificados de acordo com o Índice de Eichner (IE)^(12)^. O Índice de Eichner é um método de classificação de perdas dentárias baseado no contato oclusal entre dentes existentes nas regiões pré-molares e molares. Assim, o IE foi determinado pelos componentes de contato vertical existentes entre os molares bilaterais e categorizados em três tipos: Classe A, contato entre quatro zonas de suporte oclusal, Classe B, contato entre uma a três zonas de suporte oclusal e Classe C, sem contato oclusal. Além da descrição de uso de próteses dentárias, o IE foi avaliado utilizando o suporte oclusal habitual para mastigação, ou seja, com a utilização da reabilitação protética existente.

A mobilidade e força de língua foram aspectos subjetivos avaliados pelo fonoaudiólogo, que solicitou que o paciente executasse os movimentos desejados de protrusão e lateralização com a língua, e a protrusão de língua contra a resistência do dedo enluvado. Foram adotados como critérios de normalidade a capacidade de executar corretamente os comandos desejados e manter a força isométrica sobre a resistência do dedo. A fraqueza lingual foi observada quando o avaliador solicitava que o paciente utilizasse a força máxima voluntária de língua contra a resistência do dedo enluvado do próprio avaliador, que apesar de serem medidas qualitativas, a depender da experiência prévia de comparação da normalidade, a fraqueza resulta em uma baixa duração de contração muscular e diminuição rápida do movimento isométrico^(13)^.

Com relação a função de fonação, o avaliador ainda foi o responsável por solicitar ao paciente que emitisse o som da vogal “a” pelo máximo de tempo possível após modelo, adotando-se como critérios de normalidade o tempo cronometrado de 14s para mulheres e 20s para homens, para a medida do Tempo Máximo de Fonação (TMF)^(14)^. Nesse momento, foi possível realizar a análise perceptivoauditiva da voz e anotar a presença/ausência de rugosidade durante a emissão da vogal. Solicitou-se também a emissão de uma tosse espontânea forte para avaliar a eficiência subjetiva na produção da tosse sob comando (eficiente/fraca) para uma eventual limpeza faríngea. Todas as alterações foram descritas e anotadas para dar prosseguimento a avaliação instrumental da deglutição.

O exame da videoendoscopia da deglutição foi realizado por um médico residente, acompanhado por um otorrinolaringologista responsável e pelo fonoaudiólogo com experiência em disfagia orofaríngea, em consonância com o protocolo da instituição. Foi utilizado um nasofibroscópio flexível, marca Olympus® de 3,2mm de diâmetro, modelo LF-P com microcâmera e fonte de luz acoplados. O paciente foi orientado a permanecer na posição sentada durante toda realização do exame, não sendo utilizado anestésico tópico durante a introdução do instrumento na cavidade nasal até a região de hipofaringe. A sensibilidade faríngea foi verificada com o toque do nasofibroscópio em região de epiglote e observada reação de constricção faríngea. Após as condutas de análise das estruturas, realizadas pelo médico, o fonoaudiólogo ofertava alimentos, saborizados artificialmente com suco dietético em pó, corados artificialmente com anilina azul e espessados por meio de um produto instantâneo a base de amido de milho, além de, ao final, ser ofertada uma porção de biscoito salgado de 8g por demanda livre.

As consistências alimentares avaliadas de acordo com a classificação *International Dysphagia Diet Standartisation Initiative* (IDDSI)^(15)^ seguiram a ordem: nível 2 (líquido levemente espessado), nível 4 (líquido extremamente espessado) e nível 0 (líquido fino) em três ofertas em uma colher metálica de 5 mL, enquanto o nível 7 (sólido normal) foi uma oferta de porção única.

Os três profissionais anteriormente citados, com experiência na realização do exame, foram responsáveis por interpretar, avaliar simultaneamente, por consenso a presença de deglutições múltiplas, escape oral posterior e resíduos faríngeos em região de valéculas e/ou seios piriformes, de acordo com a escala *Yale Pharyngeal Residue Severity Rating Scale* (YPRSRS)^(16)^ (1 - Nenhum, 2 - Vestígio faríngeo, 3 - Resíduo leve, 4 - Resíduo moderado, 5 - Resíduo grave). Também foi avaliada a presença de penetração e aspiração laríngea. Foram considerados para a análise, a partir da primeira oferta, os seguintes parâmetros: deglutições múltiplas, considerada a partir de mais de duas tentativas de deglutir a mesma oferta^(17,18)^; escape oral posterior, pela presença do escape prematuro do alimento em região valecular antes de desencadear a reação de deglutição^(17,18)^; resíduo faríngeo, por meio da identificação de presença residual de alimento corado em região de valéculas e/ou seios piriformes, após a deglutição da primeira oferta em diante^(17,18)^; penetração laríngea, via observação de presença residual de alimento corado em região de pregas vocais^(17,18)^; e aspiração laríngea, quando houvesse resíduo de alimento corado abaixo das pregas vocais^(17,18)^. Toda análise aconteceu em tempo real e as imagens foram armazenadas em um computador do próprio ambulatório, para serem revisadas quantas vezes os profissionais julgassem necessárias após a realização do exame.

O nível de ingestão oral foi avaliado pelos profissionais após a realização do exame. Foi utilizada a escala *Functional Oral Intake Scale* (FOIS)^(19)^ baseada na análise do exame, existência e necessidade de espessamento de líquidos. As pontuações na escala FOIS variam de 1 (nada por via oral) a 7 (ingestão total por via oral sem restrições). Enquanto que, para avaliar o risco nutricional, uma nutricionista do serviço foi responsável por aplicar o instrumento *Malnutrition Screening Tool* (MST)^(20)^, traduzido e adaptado para o português, constituído por três perguntas com relação a autopercepção de perda de peso e perda de apetite para se alimentar no último mês. O MST é um instrumento acessível e rápido de se aplicar em adultos na admissão hospitalar, em que, valores iguais ou superiores a dois representam risco nutricional e necessidade de avaliação nutricional detalhada.

### Análise dos dados

Para análise dos dados, foi utilizada a estatística descritiva com medidas de tendência central e de dispersão, frequências absolutas e relativas. Foi utilizado o teste Shapiro-Wilk para verificar a normalidade da distribuição das variáveis quantitativas dependentes. Para análise inferencial, foi aplicado o teste Mann-Whitney na comparação dos protocolos utilizados, o Qui-quadrado de Pearson ou Exato de Fisher para analisar as variáveis categóricas, como “avaliação fonoaudiológica” e “sinais faríngeos videoendoscópios”, a depender da frequência esperada por cada célula ser superior ou igual a 5. Além disso, foi utilizada a correlação bivariada de Spearman com regressão linear simples para obter o coeficiente de determinação entre as variáveis quantitativas na amostra. Para todas as análises considerou-se o nível de significância de 5%.

## RESULTADOS

A amostra foi dividida em dois grupos de acordo a autopercepção de queixas de deglutição, sendo o primeiro grupo com 44 idosos com queixas de dificuldade em deglutir e média de 71,5 (±8,7) anos, e o segundo grupo com 27 idosos sem queixas de dificuldades em deglutir e média de 67,9 (±5,3) anos. As características e comparações dos grupos como gênero, idade, presença de hipertensão arterial sistêmica, diabetes mellitus e uso de polimedicamentos estão descritas na Tabela 1. Não houve diferença com relação a presença dos diagnósticos e uso de polimedicamentos entre os grupos.

**Tabela 1:** Comparação entre gênero, idade, hipertensão arterial sistêmica, diabetes mellitus e polimedicamentos entre os grupos

Variáveis	Grupos		Valor de p
	Idosos com queixas	Idosos sem queixas	
	n= 44 (%)	n= 27 (%)	
Gênero			
Feminino	28 (63,6)	16 (59,3)	0,712
Masculino	16 (36,4)	11 (40,7)	
Idade (anos)	71,5 (±8,71)	67,9 (±5,39)	-
Hipertensão Arterial Sistêmica			
Sim	14 (31,8)	8 (29,6)	0,846
Não	30 (68,2)	19 (70,4)	
Diabetes Mellitus			
Sim	5 (11,4)	3 (11,1)	0,944
Não	39 (88,6)	24 (88,9)	
Uso de polimedicamentos			
Sim	8 (18,2)	3 (11,1)	0,424
Não	36 (81,8)	24 (88,9)	

Todos os dados são expressos em número (%) ou média (desvio padrão)

A relação dos achados da avaliação fonoaudiológica entre os grupos foi descrita na Tabela 2. Idosos com queixas apresentaram diferenças, como força da língua reduzida, tempo máximo de fonação reduzido e presença de rugosidade na emissão da vogal. Com relação ao estado oral, o grupo de idosos com queixas apresentou diferenças no uso de próteses dentárias e na oclusão habitual classificada em classe B ou C do IE, em comparação ao grupo sem queixas.

**Tabela 2:** Comparação entre os achados fonoaudiológicos e o estado oral entre os grupos

Achados fonoaudiológicos	Grupos		Valor de p
	Idosos com queixas	Idosos sem queixas	
	n= 44 (%)	n= 27 (%)	
Mobilidade de língua			
Preservada	35 (79,5)	25 (92,6)	0,187
Reduzida	9 (20,5)	2 (7,4)	
Força de língua			
Preservada	30 (68,2)	25 (92,6)	0,020**
Reduzida	14 (31,8)	2 (7,4)	
Tempo de trânsito oral			
Adequado	41 (93,2)	26 (96,3)	0,581
Aumentado	3 (6,8)	1 (3,7)	
Tempo máximo de fonação			
Adequado	32 (72,7)	25 (11,1)	**0,050** **
Reduzido	12 (27,3)	2 (88,9)	
Rugosidade			
Ausente	20 (45,5)	20 (74,1)	**0,018** *
Presente	24 (54,5)	7 (25,9)	
Tosse espontânea			
Eficiente	37 (84,1)	26 (96,3)	0,114
Fraca	7 (15,9)	1 (3,7)	
**Estado oral**			
Uso de próteses dentárias			
Sim	8 (18,2)	12 (44,4)	0,017*
Não	36 (81,8)	15 (55,6)	
Índice de Eichner			
Classe A	23 (52,3)	21 (77,8)	0,032*
Classe B ou C	21 (47,7)	6 (22,2)	
Estase salivar			
Ausente	42 (95,5)	27 (100)	0,522
Presente	2 (4,5)	0 (0,0)	

* Qui-quadrado de Pearson;

** Exato de Fisher

Todos os dados são expressos em número (%)

Na relação dos sinais faríngeos da videoendoscopia da deglutição, apresentada na Tabela 3, verificou-se diferença nas ocorrências de escape oral posterior e resíduos faríngeos nas consistências de nível 0, 2 e 4. Assim como houve diferença nas ocorrências de penetração laríngea na consistência de nível 0. Nota-se que o grupo de idosos com queixas foi o único a apresentar sinais de penetração e aspiração laríngea nas consistências de líquidos espessados (nível 2 e 4). Não houve ocorrências de sinais faríngeos na consistência de sólido normal (nível 7) no grupo de idosos sem queixas.

**Tabela 3:** Relação entre os sinais faríngeos da videoendoscopia da deglutição por consistência alimentar entre os grupos

Sinais faríngeos por nível de consistência alimentar (IDDSI)	Grupos		Valor de p
	Idosos com queixas	Idosos sem queixas	
	n= 44 (%)	n= 27 (%)	
Sensibilidade faríngea			
Preservada	36 (81,8)	24 (88,9)	0,515
Reduzida	8 (18,2)	3 (11,1)	
Fechamento glótico			
Completo	40 (90,9)	27 (100)	0,290
Incompleto	4 (9,1)	0 (0,0)	
Líquido fino (nível 0)			
Deglutições múltiplas			
Ausente	41 (93,2)	26 (96,3)	0,581
Presente	3 (6,8)	1 (3,7)	
Escape oral posterior			
Ausente	21 (47,7)	20 (74,1)	0,029*
Presente	23 (52,3)	7 (25,9)	
Resíduos faríngeos			
Ausente	21 (47,7)	20 (74,1)	**0,029** *
Presente	23 (52,3)	7 (25,9)	
Penetração laríngea			
Ausente	37 (84,1)	27 (100)	**0,039** **
Presente	7 (15,9)	0 (0,0)	
Aspiração laríngea			
Ausente	42 (95,5)	27 (100)	0,522
Presente	2 (4,5)	0 (0,0)	
Líquido levemente espessado (nível 2)			
Deglutições múltiplas			
Ausente	41 (93,2)	27 (100)	0,283
Presente	3 (6,8)	0 (0,0)	
Escape oral posterior			
Ausente	22 (50,0)	21 (77,8)	0,020*
Presente	22 (50,0)	6 (22,2)	
Resíduos faríngeos			
Ausente	24 (54,5)	22 (81,5)	0,021*
Presente	20 (45,5)	5 (18,5)	
Penetração laríngea			
Ausente	41 (93,2)	27 (100)	0,283
Presente	3 (6,8)	0 (0,0)	
Aspiração laríngea			
Ausente	43 (97,7)	27 (100)	0,430
Presente	1 (2,3)	0 (0,0)	
Líquido extremamente espessado (nível 4)			
Deglutições múltiplas			
Ausente	41 (93,2)	27 (100)	0,283
Presente	3 (6,8)	0 (0,0)	
Escape oral posterior			
Ausente	23 (52,3)	23 (85,2)	0,005**
Presente	21 (47,7)	4 (14,8)	
Resíduos faríngeos			
Ausente	22 (50,0)	24 (88,9)	<0,001**
Presente	22 (50,0)	3 (11,1)	
Penetração laríngea			
Ausente	43 (97,7)	27 (100)	0,430
Presente	1 (2,3)	0 (0,0)	
Aspiração laríngea			
Ausente	44 (100)	27 (100)	-
Presente	0 (0,0)	0 (0,0)	
Sólido normal (nível 7)			
Deglutições múltiplas			
Ausente	44 (100)	27 (100)	-
Presente	0 (0,0)	0 (0,0)	
Escape oral posterior			
Ausente	39 (88,6)	27 (100)	0,149
Presente	5 (11,4)	0 (0,0)	
Resíduos faríngeos			
Ausente	38 (86,4)	27 (100)	0,076
Presente	6 (13,6)	0 (0,0)	
Penetração laríngea			
Ausente	44 (100)	27 (100)	-
Presente	0 (0,0)	0 (0,0)	
Aspiração laríngea			
Ausente	44 (100)	27 (100)	-
Presente	0 (0,0)	0 (0,0)	

* Qui-quadrado de Pearson;

** Exato de Fisher

Legenda: IDDSI = International Dysphagia Diet Standardisation Initiative

Todos os dados são expressos em número (%)

Ao comparar a igualdade das medianas dos protocolos de classificação dos resíduos faríngeos (YPRSRS), nível de ingestão por via oral (FOIS) e risco nutricional (MST), o grupo de idosos com queixas apresentou diferenças significativas em comparação ao grupo sem queixas (Tabela 4). À medida que o grupo de idosos com queixas apresentou, em sua maioria, resíduos classificados em grau de vestígio (YPRSRS 2), o grupo de idosos sem queixas apresentou nenhum resíduo (YPRSRS 1) em valéculas e seios piriformes.

**Tabela 4:** Comparação entre a gravidade dos resíduos faríngeos, risco nutricional e o nível de ingestão por via oral entre os grupos

	Grupos		Valor de p
	Idosos com queixas	Idosos sem queixas	
YPRSRS	2 (1-3)	1 (1-1)	**0,011** *
MST	0 (0-1,2)	0 (0-0)	0,009*
FOIS	5,5 (5-6,2)	7 (7-7)	<0,001*

* U de Mann-Whitney

Legenda: YPRSRS = Yale Pharyngeal Residue Severity Rating Scale; MST = Malnutrition Screening Tool; FOIS = Functional Oral Intake Scale

Todos os dados são expressos em mediana (intervalo interquartil 25-75)

Na Tabela 5 encontram-se os resultados da análise de correlação de Spearman entre a escala FOIS e os protocolos YPRSRS e MST na amostra. Houve correlação negativa moderada com o YPRSRS e o MST. O coeficiente de determinação indicou que a gravidade dos resíduos faríngeos e o risco nutricional foi responsável por 30% e 38%, respectivamente, de influência no nível de ingestão por via oral nos indivíduos da amostra. O cálculo da fórmula para obter a previsão do nível de ingestão por via oral pode ser representado por: 7,15 * (-0,60) * (valor de YPRSRS); 6,39 * (-0,73) * (valor de MST).

**Tabela 5:** Correlação e regressão linear entre o nível de ingestão oral com a gravidade dos resíduos faríngeos e o risco nutricional na amostra

Gravidade dos resíduos faríngeos (YPRSRS) e o risco nutricional (MST)	FOIS		
	⍴	Valor de p	R^2^
YPRSRS	-0,526	<0,001	0,305
MST	-0,622	<0,001	0,387

**Legenda:** YPRSRS = Yale Pharyngeal Residue Severity Rating Scale; MST = Malnutrition Screening Tool; FOIS = Functional Oral Intake Scale; R^2^ = Coeficiente de determinação; Correlação de Spearman e Regressão Linear Simples

## DISCUSSÃO

No presente estudo os resultados sugerem que o grupo de idosos com queixas apresentou diferenças com redução na força de língua, tempo máximo de fonação e presença de rugosidade na emissão, além de escape oral posterior e resíduos faríngeos nas consistências de nível 0, 2 e 4, e penetração laríngea na consistência de nível 0. Foi possível verificar correlação negativa moderada entre o nível de ingestão oral com a gravidade dos resíduos faríngeos e o risco nutricional na amostra.

Nesse contexto, as alterações funcionais na fase oral atribuídas ao envelhecimento foram investigadas anteriormente com predomínio de estudos com foco na mobilidade e força de língua, pois desempenha papel fundamental na captação, pressão, transporte e propulsão do bolo alimentar da cavidade oral para fase faríngea^(21)^. Os resultados do estudo sugerem que a força da língua estaria reduzida para as tarefas que ela poderia desempenhar na deglutição e que, consequentemente, poderia afetar na sua eficiência, apesar de ser medida antes da atividade propriamente dita. Com isso, o achado corrobora outros estudos que encontraram alterações na musculatura lingual relacionadas à idade, com relevante redução subsequente na pressão intraoral^(21,22)^, entretanto, sem dados sobre a redução da atividade lingual durante a atividade em si.

Com relação a função de fonação, foram parâmetros pesquisados para conferir informações relevantes sobre a coaptação das pregas vocais, bem como alterações de origem orgânica na laringe que poderiam reduzir a capacidade de proteção das vias aéreas inferiores durante a deglutição. O tempo máximo de fonação reduzido no grupo de idosos com queixas pode estar relacionado a baixa capacidade de proteção e presença de penetração de alimento nas pregas vocais, evidenciados na consistência de líquido ralo (nível 0). Em idosos, há evidências que a atividade dos fibroblastos e a produção do ácido hialurônico diminuem^(9)^. Como consequência, a rigidez do ligamento vocal aumenta, e o aumento da concentração de colágeno reduz as propriedades viscoelásticas da mucosa, o que pode resultar em rugosidade na análise perceptivoauditivo da voz^(9)^.

Em referência ao estado oral, o grupo de idosos com queixas apresentou diferença no uso de próteses dentárias e no contato oclusal entre zonas de molares. O grupo em estudo apresentou menos uso de próteses dentárias na mesma medida que apresentou classe B ou C do IE, ou seja, houve ausências dentárias significativas em zonas de contato oclusal entre molares durante a mastigação habitual sem possibilidade de uso de reabilitação oral. As consequências das ausências dentárias em idosos são amplamente pesquisadas e conferem uma perspectiva de que o número de dentes reduzidos está relacionado a déficit na eficiência mastigatória, tempo maior de processamento do alimento e seleção de alimentos mais fáceis de mastigar^(23)^. Além do mais, a prevalência de cárie radicular em pessoas com idade superior a 60 anos é duas vezes maior do que em jovens, o que pode tornar a estrutura neuromuscular da polpa sensível, dolorosa e até propensa a infecções^(24)^. Isso pode levar a uma redução na mastigação e ingestão de vegetais, frutas e fibras e, por consequência, estar propenso a perda de peso relevante com risco nutricional^(25)^.

A análise dos sinais faríngeos foi realizada por meio da VED com quatro consistências alimentares distintas. O grupo de idosos com queixas apresentou diferenças nos sinais de escape oral posterior e resíduos faríngeos nos líquidos (nível 0, 2 e 4), enquanto que a penetração laríngea ocorreu na consistência de líquido fino (nível 0). Como não houve diferença nos sinais de disfagia na consistência sólida, as consistências mais fáceis de fluir foram as mais inseguras para ao grupo em estudo, principalmente, o líquido sem espessamento. Em outro estudo, cerca de 55% dos idosos apresentaram sinais de penetração no líquido durante a deglutição, na mesma proporção que encontraram redução no reflexo de tosse^(26)^. Apesar de haver sinais de comprometimento na eficiência da deglutição, a análise dos resultados sugere que os sinais considerados mais graves de disfagia foram preponderantes para o grupo com queixas, visto que a permanência recorrente de resíduos faríngeos e a possibilidade de uma eventual penetração desse material após a deglutição pode ser preditor para as queixas clínicas^(27)^. Caso não seja efetivamente eliminado na faringe com reações de proteção como tosse e pigarro, o líquido pode se tornar desafiador no cotidiano do indivíduo.

Quando comparamos os valores de mediana dos protocolos utilizados, observamos diferenças no nível de ingestão oral, grau dos resíduos faríngeos e o risco nutricional entre os grupos. Nesse sentido, a gravidade dos resíduos foi relevante para o grupo com queixas, que apresentou na mesma proporção força de língua reduzida e penetração laríngea. Isso demonstra uma cadeia de comprometimentos no prosseguimento das fases da deglutição, em comparação ao grupo sem queixas. A presença de resíduos após a deglutição, bem como sua gravidade de acúmulo nos recessos faríngeos, estão relacionados a redução na atividade muscular da língua e à redução no movimento hiolaríngeo, conforme observado em um estudo anterior com adultos disfágicos^(28)^. Por esse motivo, ainda que a maioria dos indivíduos no grupo com queixas estivessem sem riscos nutricionais, a amplitude do intervalo interquartil foi maior, o que denota alguns indivíduos em risco nutricional advindo dos comprometimentos da deglutição. Como os indivíduos da amostra são caracterizados como idosos da comunidade que procuraram atendimento, o risco nutricional estaria atenuado pelas modificações alimentares e redução de sua ingestão, visto que em idosos hospitalizados o risco nutricional representa 51%^(25,27)^.

Na amostra, os resultados indicaram que a gravidade dos resíduos e o risco nutricional estão correlacionados e inversamente proporcionais à ingestão por via oral. Quanto menor a possibilidade de ingestão por via oral, maior será o grau dos resíduos faríngeos e o risco nutricional. Há fortes evidências que o resíduo faríngeo seja preditor para eventos de penetração e aspiração em pacientes disfágicos^(28)^. Contudo, os líquidos finos e levemente espessados são as consistências que apresentam maior frequência de penetração laríngea, em comparação às demais consistências^(29)^. Mesmo que pareça tentador pensar que os líquidos espessados sejam mais seguros, deve-se notar que o volume ofertado na colher pode ser um fator de confusão entre o momento da avaliação (5 mL) e o consumo no cotidiano do indivíduo.

Outro dado que devemos analisar é o aumento do risco nutricional à medida que diminui o nível de ingestão por via oral. Ao confirmar que o risco nutricional está relacionado aos transtornos de deglutição, podemos levantar a hipótese de que os idosos da amostra possam restringir o consumo de determinados alimentos devido à dificuldade na ingestão, além de sofrerem uma eventual perda de apetite identificadas no rastreio nutricional. Sabe-se que o processamento e liquidificação de alimentos sem aconselhamento nutricional adequado pode resultar na diminuição da ingestão calórica, estado nutricional e apetite^(30)^. Tanto a desnutrição quanto a desidratação podem ser causadas pela disfagia, mas também pioram os transtornos de deglutição^(30,31)^. Nesse efeito cíclico, vale ressaltar a importância do acompanhamento e otimização do estado nutricional para manejo correto da disfagia no idoso.

Como limitações do estudo podemos citar a ausência de um instrumento validado para o rastreamento da disfagia no grupo com queixas, entretanto, visto que os participantes da pesquisa foram encaminhados com sintomas para realizar a investigação objetiva da deglutição, isso pode dispensar a utilização de um rastreio. É importante mencionar a ausência da avaliação instrumental da força da língua, o número desigual de participantes em cada grupo, a falta de informações sobre o histórico ocupacional e exposição a agentes químicos, bem como a ausência de dados complementares sobre o estado nutricional, como o recordatório alimentar. Entre os pontos fortes do estudo, destacam-se a criteriosa seleção da amostra, a análise subjetiva e instrumental da deglutição, assim como a avaliação do nível de ingestão oral e do risco nutricional entre os grupos. Esses elementos permitiram a elaboração de novas hipóteses de pesquisa sobre o momento em que a dificuldade de deglutição se torna uma queixa, além de proporcionar novas perspectivas sobre a disfagia em idosos aparentemente saudáveis.

## CONCLUSÃO

O grupo de idosos com queixas apresentou diferenças com redução na força de língua, tempo máximo de fonação reduzido e presença de rugosidade na emissão, além de escape oral posterior e resíduos faríngeos nas consistências alimentares de nível 0, 2 e 4, e penetração laríngea na consistência de nível 0. A correlação indicou que, quanto menor o nível de ingestão oral, maior a gravidade dos resíduos faríngeos e o risco nutricional na amostra.
